# Comparison of the transcriptomes of two tardigrades with different hatching coordination

**DOI:** 10.1186/s12861-019-0205-9

**Published:** 2019-12-09

**Authors:** Yuki Yoshida, Kenta Sugiura, Masaru Tomita, Midori Matsumoto, Kazuharu Arakawa

**Affiliations:** 1 Institute for Advanced Biosciences, Keio University, Mizukami 246-2, Kakuganji, Tsuruoka, Yamagata, 997-0052 Japan; 2 Systems Biology Program, Graduate School of Media and Governance, Keio University, Fujisawa, Kanagawa Japan; 3 Graduate School of Science and Technology, Keio University, Fujisawa, Yokohama Japan

**Keywords:** Tardigrades, Developmental stages, RNA-Seq, Ecdysone

## Abstract

**Background:**

Tardigrades are microscopic organisms, famous for their tolerance against extreme environments. The establishment of rearing systems of multiple species has allowed for comparison of tardigrade physiology, in particular in embryogenesis. Interestingly, in-lab cultures of limnic species showed smaller variation in hatching timing than terrestrial species, suggesting a hatching regulation mechanism acquired by adaptation to their habitat.

**Results:**

To this end, we screened for coordinated gene expression during the development of two species of tardigrades, *Hypsibius exemplaris* and *Ramazzottius varieornatus*, and observed induction of the arthropod molting pathway*.* Exposure of ecdysteroids and juvenile hormone analog affected egg hatching but not embryonic development in only the limnic *H. exemplaris*.

**Conclusion:**

These observations suggest a hatching regulation mechanism by the molting pathway in *H. exemplaris*.

## Background

Tardigrades are microscopic animals found in various meiofauna and are prominently known for their capability to tolerate various extreme environments during the ametabolic state designated as cryptobiosis [23]. Recent advances in the establishment of tardigrade genomic information has allowed for comprehensive molecular analysis in tardigrade species [17, 41, 47, 48].

The establishment of rearing systems for several species has enabled various quantitative zoological and molecular analyses, mainly focused on the limnic *Hypsibius exemplaris* and terrestrial *Ramazzottius varieornatus* [14, 18]. In particular, developmental research of the *H. exemplaris* embryo has provided valuable evidence regarding the evolution and phylogeny of tardigrades within Ecdysozoa (Reviewed in Altiero et al. [3]). A large amount of developmental research has focused on the morphology of *H. exemplaris*, due to their rapid embryogenesis and life cycle. The *H. exemplaris* embryo hatches after around 4–4.5 days [14]. Observations based on microscopy-based techniques have reported that the first cleavage is observed at 2 h post laying (hpl), and gastrulation is completed by 16.5 hpl [14]. Further Scanning Electron Microscope (SEM) observations indicate that the head and limb buds, segmental lines, claws and mouth are visible by − 25 hpl, − 30 hpl and 35 and 50 hpl, respectively [16], suggesting that embryonic development is concluded around 3 days after oviposition. On the other hand, establishment of gene sequence databases and genetic engineering methods has enabled the molecular analysis of development related genes [9, 17, 48]. Immunostaining-based expression analysis and in situ hybridization analysis has enabled the analysis of expression patterns of several genes: pair-rule gene *paired* (*Pax3/7*), the segment polarity gene *engrailed* [13], and Homeobox genes [39]. Additionally, RNA interference knock-down of the expression of *mago-nashi* resulted in failure of elongation in the developing embryo, suggesting a major role of *mago-nashi* during embryogenesis [43].

On the other hand, the hatching timing of in-lab cultures of *R. varieornatus* extended to 5.8 ± 1.2 days (mean ± SD), slightly longer than *H. exemplaris* [18]. Relatively higher diversity in hatching timing of in-lab cultures which lack non-predictable stimuli (i.e. environmental factors) suggests that the diversity may be the result of adaptation to their living habitat.

To this end, we sought to identify factors that cause diversity in hatching timing. We have previously analyzed the genomes of two tardigrades from different habitats, the limnic *H. exemplaris* and terrestrial *R. varieornatus.* Genomes of both species have been sequenced, making these two species ideal for a comparative genomic/transcriptomic analysis, such as the identification of anhydrobiosis related genes [17, 48]. We first observed hatching timing in *H. exemplaris* to calculate the diversity in this species. We then conducted a comparative transcriptomic analysis of the embryo and juvenile stages in both species to identify pathways that are induced around 1 day prior to hatching. We finally exposed embryos to the ecdysteroid 20-Hydroxyecdysone (20-E) and juvenile hormone analog Fenoxycarb to understand the effects of such substances on the embryo. We observed that *H. exemplaris* also has tightly regulated embryo hatching, and the arthropod molting pathway was induced. 20-E and Fenoxycarb exposure induced a latent state in only the *H. exemplaris* embryo. These findings support the idea that limnic species may have tighter regulation in embryo hatching and that embryo hatching may be hormone regulated.

## Results

**Fig. 1:**
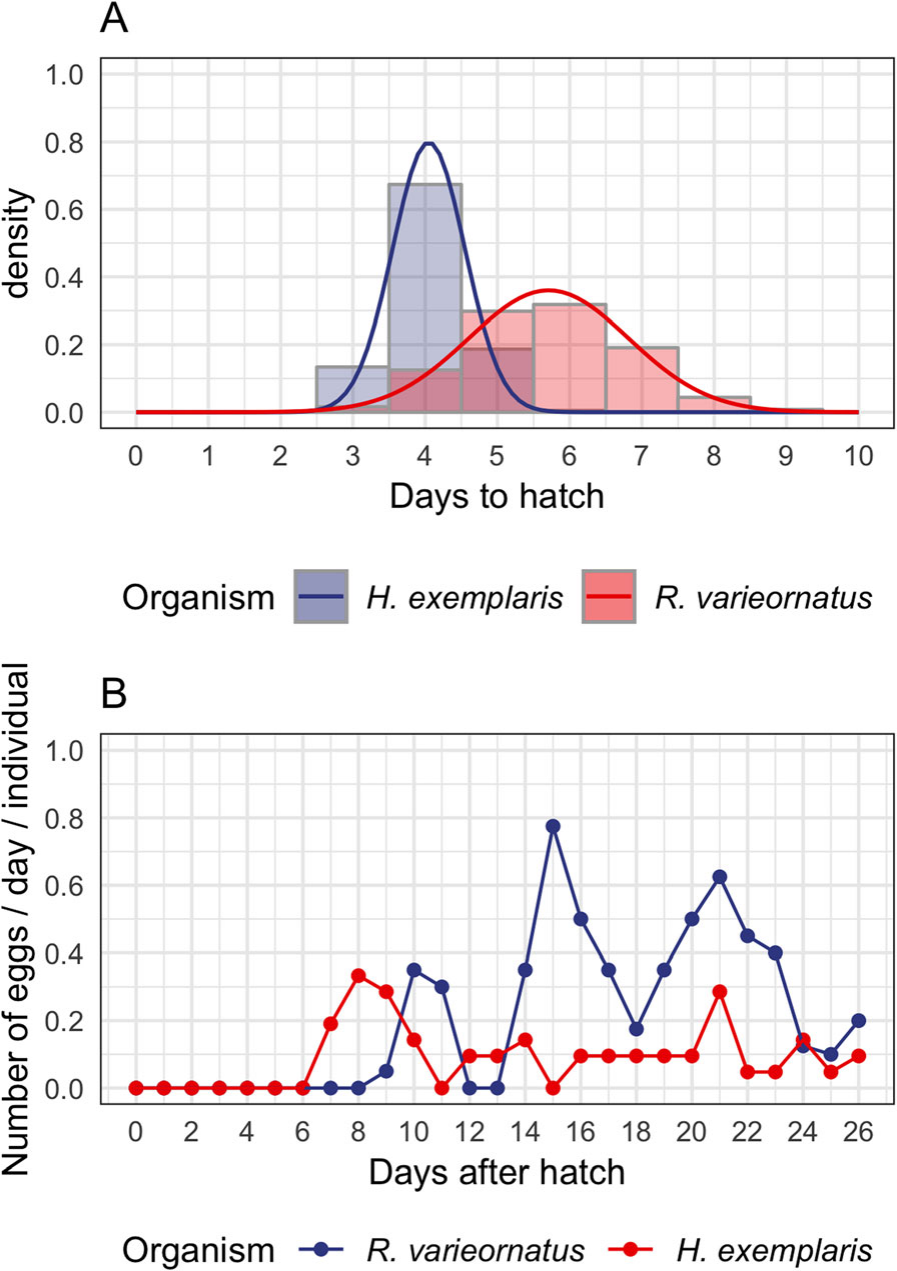
Comparison of hatching timing between *H. exemplaris* and *R. varieornatus.* **a** Probability density plot of the days required for egg hatching in both species. Seventy-three and 259 embryos were observed for *H. exemplaris* and *R. varieornatus*, respectively. A normal distribution curve is overlaid for each species. **b** Number of eggs that were oviposit in each day per individual in *H. exemplaris* and *R. varieornatus*. Twenty-one and 40 individuals were observed for each species, respectively. Data from Horikawa et al. [18] was used for *R. varieornatus*

Previous studies have shown that embryonic development occurs in 4–4.5 days in *H. exemplaris*, and generation time takes 13–14 days. We collected embryos immediately after oviposition, and observed the development time, as well as generation time (Fig. 1a). Embryonic development took 4.03 ± 0.50 days from oviposition to hatch. The 1st and 2nd oviposition occurred around 7–11 days and 16–22 days after hatching, with 2.68 ± 0.75 and 8.34 ± 4.12 eggs per individual, respectively (Fig. 1b). The body length reached the average length of an adult (240 μm) at around 15d after oviposition (Additional file 1: Figure S1). In comparison, *R. varieornatus* requires 5.72 ± 1.13 days to hatch and the 1st and 2nd oviposition occurs at 8–12 days and 13–18 days after hatching. Additionally, we observed that the average number of eggs per individual was 7.37 ± 2.83, similar to 7.8 in previous studies [18]. We therefore assume that hatching related genes may be upregulated around Egg 3d and 5d in *H. exemplaris* and *R. varieornatus*, respectively.

**Fig. 2:**
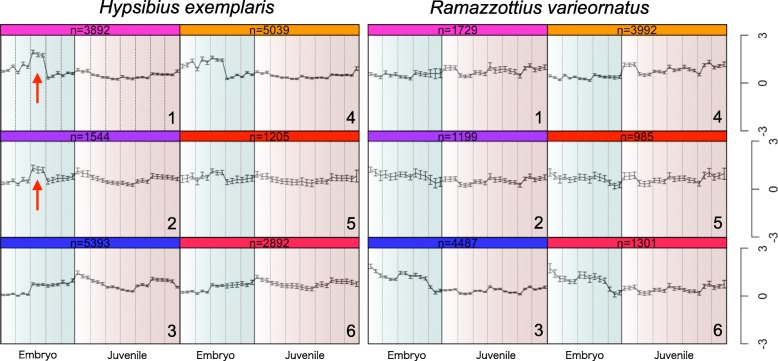
SOM clustering of gene expression profiles during development. SOM clustering of TPM values were performed in R. Genes with TPM values over 1 were used. The areas colored in blue and red represent embryo and juvenile stages, respectively. The Y axis indicates Z-scale normalized TPM values. The arrows indicate an increase in Egg 3d of *H. exemplaris*

We have previously sequenced the transcriptome of the embryo and juvenile stages in *H. exemplaris*, and, in part, *R. varieornatus*. Hence, we additionally sequenced the juvenile stages of *R. varieornatus*, and conducted a comprehensive analysis of developmental and juvenile stages of both species (Additional file 9: Table S1). High correlation was observed between replicates (*N* = 3). In order to validate our sequencing data, we first compared the expression profiles of HOX genes observed previously by single embryo RNA-Seq methods [26]. In our data, HOX genes showed an Egg 2-3d specific expression in *H. exemplaris*, while a wide expression was observed at the Egg 2-4d in *R. varieornatus* (Additional file 2: Figure S2). In particular, *caudal* was expressed in Egg 2d, while other HOX genes were expressed at Egg 2-3d. In comparison, *caudal* was mainly expressed around 500 min (8.3 h), while other HOX genes (i.e. *lab*, *ftz*, *disformed*, *zen*) were expressed around 1600 min (26.7 h) in the CEL-Seq data (Additional file 3: Figure S3). To obtain a general view of the transcriptome profile of both species, we conducted SOM clustering of gene expression profiles. We found two out of six clusters showed an Egg 3d-specific expression in *H. exemplaris* (Fig. 2, groups 1 and 2, Additional file 10: Data S1), suggesting dynamic shift in the embryonic transcriptome prior to hatching in only *H. exemplaris*. No such shift was observed in *R. varieornatus*.

**Fig. 3:**
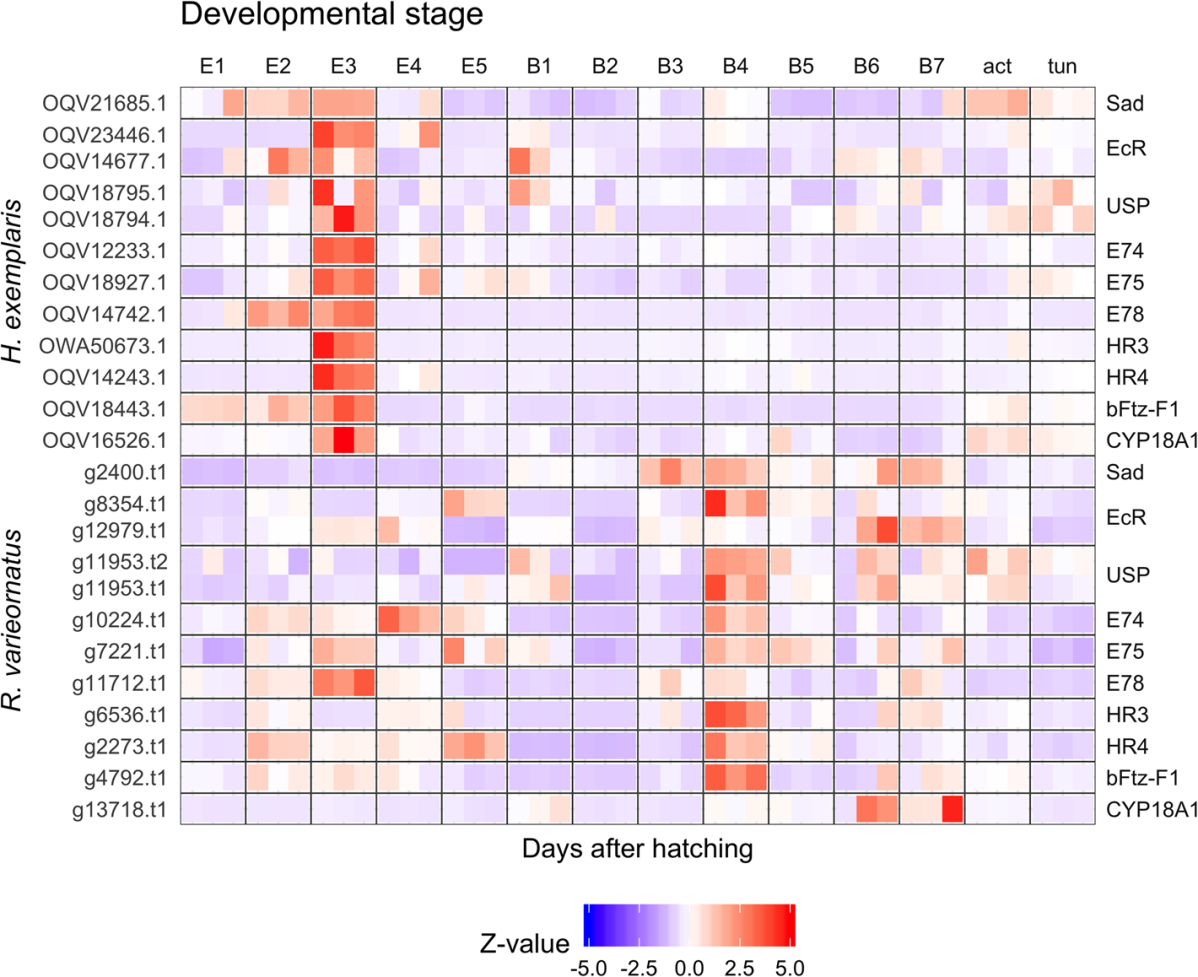
Gene expression patterns for molting pathways during development. Genes identified in Schumann et al. [37] were identified by BLASTP searches against amino acid sequences of *H. exemplaris* and *R. varieornatus*, and Z-scaled TPM values were visualized. E: Egg, B: Juvenile, act/tun: Adult stages. The identified orthologs are as follows; *sad* (OQV21685.1, g2400.t1), *EcR* (OQV14677.1, OQV23446.1, g12979.t1, g8354.t1), *USP/RXR* (OQV18794.1, OQV18795.1, g11953.t1, g11953.t2), *E74* (OQV12233.1, g10224.t1), *E75* (OQV18927.1, g7221.t1), *E78* (OQV14742.1, g11712.t1), *HR3* (OWA50673.1, g6536.t1), *HR4* (OQV14243.1, g2273.t1), *bFtz-F1* (OQV18443.1, g4792.t1), *CYP18A1* (OQV16526.1, g13718.t1). An additional copy of *EcR* was found during this analysis (*H. exemplaris* OQV23446.1, *R. varieornatus* g8354.t1)

In order to identity potential mediators of hatching, we screened the 5436 genes induced at the Egg 3d in *H. exemplaris* (Group 1 and 2, Additional file 11: Data S2). It is commonly known that many factors are regulated during embryonic development; however, most of these mechanisms have yet to be validated in tardigrades. We focused on signaling through neuropeptides and hormones, of which we identified 232 receptor genes in *H. exemplaris* with gene ontology annotations containing “hormone”. Eighty-two of these orthologs were contained in groups 1 and 2. We obtained 20 orthologs that were significantly expressed in only Egg 3d. Although a majority of these genes were atrial natriuretic peptide receptors, we identified multiple orthologs of the arthropod molting pathway (i.e. *EcR*, *RXR*, *E75*, *HR3*, *HR4*). Therefore, we collected other genes in the molting pathway identified in previous studies and observed their expression patterns during development. The expression patterns of these genes were highly upregulated in Egg 3d in *H. exemplaris* and constitutively moderately between Egg 2-5d in *R. varieornatus* (Fig. 3).

**Fig. 4:**
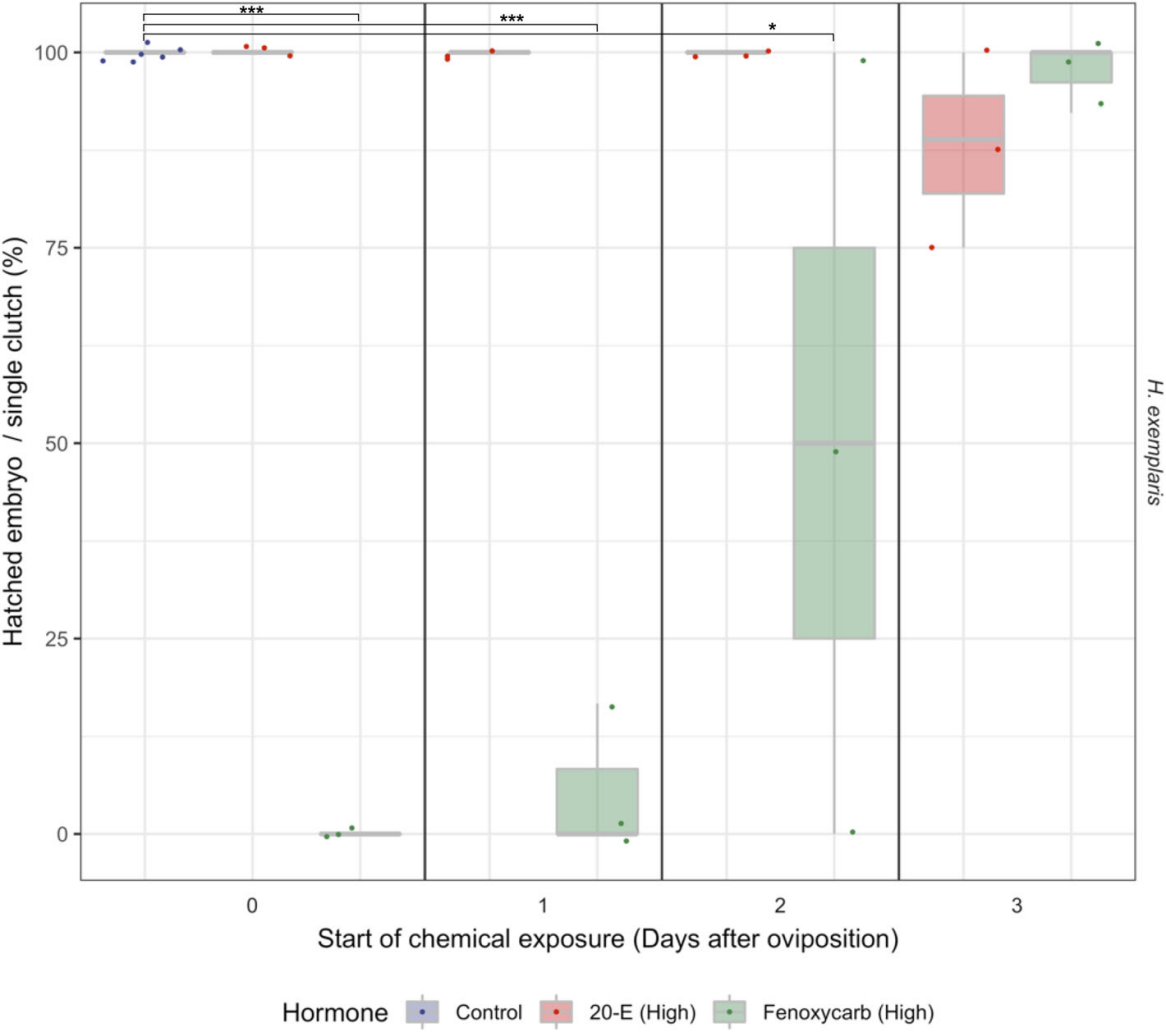
High concentration fenoxycarb exposure inhibited hatching in *H. exemplaris*. Percentages of successful hatching embryos exposed in 100 μM Fenoxycarb or 100 μM 20-E until hatch. Embryo exposed to 0.03% ethanol in Volvic mineral water at Egg 0d were used as controls. Hatching ratio for each clutch are overlaid on the boxplot. The x-axis indicates the day to start exposing the chemicals after oviposition. High concentration treatment of Fenoxycarb from 0~2 days after oviposition significantly impaired hatching, whereas treatment at 3 days after oviposition and treatment with 20-E did not. ANOVA and Tukey HSD, * = FDR < 0.05, ** = FDR < 0.01, *** = FDR < 0.001


We then validated whether disrupting the molting pathway inhibits hatching in *H. exemplaris*, we exposed *H. exemplaris* embryos to chemicals (20-E: 8.42 μM and 100 μM, Fenoxycarb: 3.3 μM and 100 μM) and observed whether they affected embryo development. While we observed that approximately 90% of the embryos exposed to low concentrations hatched within 5 days of oviposition (Additional file 6: Figure S6), there were several cases where all of the embryos in the same clutch did not hatch (Additional file 7: Figure S7). We observed claw formation at this stage, which is consistent with previous body developmental observations. Washing these embryos to remove chemical exposure induced hatching in 57.1% (4/7, five E0 embryos and two E3 embryos, of which three E0 embryos died) and 71.4% (5/7, one F0 and F1 embryos, three F2 embryo and two F3 embryos, of which one F0 and F2 embryos died) of the embryos exposed to 20-E and Fenoxycarb, respectively. Exposure of *R. varieornatus* embryo to low concentrations did not show any effect in hatching (Additional file 7: Figure S7). On the contrary, exposure to high concentrations of Fenoxycarb significantly suppressed hatching in embryos exposed at Egg 0-2d (Fig. 4, Additional file 8: Figure S8, ANOVA and Tukey HSD, FDR < 0.05). For the embryos that did not hatch, we also observed claw formation and movement inside the eggshells regardless of exposure time, suggesting that the embryos had finished development and did not die (Additional file 13: Data S4). Exposure to the EcR antagonist cucurbitacin B had no effect on embryo hatching (Data not shown).

**Additional file 13.** Movie of embryos exposed to long-term high concentration of chemicals. The shells of embryo exposed to more than 24 h to high concentration of fenoxycarb were peeled off, and movement were observed. https://github.com/abs-yy/Yoshida_and_Sugiura_etal_2018/blob/master/ Sdata_7_highConc-long-movement.mp4.


## Discussion

Previous studies on the life history of *H. exemplaris* have observed that the embryo hatches around 4–4.5 days after oviposition [14]. We have observed that the *H. exemplaris* embryo hatches in a strictly controlled manner within a short time range (4.03 ± 0.50 days) compared to the wider range observed in *R. varieornatus* (5.72 ± 1.13 day, see [18]). The small variance in *H. exemplaris* is similar to the limnic species *Isohypsibius myrops* [19]. It is worth noting that the *R. varieornatus strain* YOKOZUNA-1 was derived from a single individual [18], suggesting that genetic variation would not be higher than *H. exemplaris*, and therefore the hatching time variability is not due to the genetic variability. Therefore, we hypothesized that transcriptome regulation affects hatching timing, and factors that are related to hatching regulation may be induced around a day prior to hatching; day 3 in *H. exemplaris* and day 5 in *R. varieornatus*.

To understand the molecular pathways regulated prior to hatching, we analyzed our previously sequenced transcriptome data of the developmental stages of two tardigrade species, *H. exemplaris* and *R. varieornatus*, with newly generated data for the juvenile stages of *R. varieornatus* [9, 48]. First, we validated our sequencing data by comparison of the expression profiles of developmental genes (i.e. Homeobox genes) assayed in previous single embryo sequencing data and in-situ hybridization experimental data [26, 39]. In our data, *caudal* and *engrailed* expressed earlier than HOX orthologs, at around 24–48 h post laying, and other orthologs were expressed around 48-72 h, approximately 36 h prior to hatching. These data are similar to those of previous reports of HOX expression profiles, supporting the validity of our data. In comparison, *R. varieornatus* HOX orthologs were expressed around Egg 3d-4d, 24 h later than *H. exemplaris*. This period is also approximately 36 h prior to hatching [18]. Clustering based analysis of the embryo-juvenile time course indicated an abrupt shift in transcriptome profile at Egg 3d in *H. exemplaris*. The Egg 3d stage is immediately before the hatch in *H. exemplaris* (4.03 ± 0.53 days), which suggests that *H. exemplaris* may have some sort of trigger that drastically changes the transcriptome profile for embryo hatching. The lack of such a shift in *R. varieornatus* remains a surprise, as we have sequencing data until approximately 20 h prior to hatching. This may be due to the low quality of the sequencing data, observed by the low mapping ratio, or pathways induced in *H. exemplaris* may be expressed less abruptly, but rather gradually during the last few days of embryogenesis.

Analysis of genes induced at Egg 3d of *H. exemplaris* revealed the arthropod molting pathway was upregulated at Egg 3d. Other pathways are stated in Additional file 14 (with accompanying Additional file 4: Figure S4, Additional file 5: Figure S5 and Additional file 12: Data S3). Molting is an essential event for the development of animals in the Ecdysozoa, however, the fact that the arthropod molting pathway is induced in embryos of *Daphnia magna*, *Bombyx mori* and *Drosophila melanogaster* [11, 21, 38, 40], suggests a mechanism conserved within Arthropoda that may be related to embryo hatching. In ecdysozoans that have been investigated, the molting pathway is initiated by the signaling hormone Ecdysone, synthesized by the Halloween genes in hexapods. It is released as the active state 20-hydroxyecdysone (20-E, [20]). The released hormone binds to the ecdysone receptor (EcR) and ultraspiracle (USP) heterodimer, which regulates the expression of the early genes (HR3, HR4, E75, Ftz-F1). These respond and transmits the signal to the late response genes, leading to new cuticle biosynthesis. Although most of the downstream genes of EcR/USP were conserved, only *shadow* (*sad*) gene in the 20-E biosynthesis pathway were found in both *H. exemplaris* and *R. varieornatus* [37]. These genes were induced at Egg 3 in *H. exemplaris* and relatively constitutively expressed (most expressed at Egg 5d) in *R. varieornatus*, approximately 1 day prior to egg hatching. We did not observe a lag between early and late genes since we sampled the embryos every 24 h. Interestingly, these genes were not expressed prior to the initial molting around Juvenile 7d, whereas they were in *R. varieornatus* (Juvenile 4d). Lack of expression during molting in *H. exemplaris* may imply that this pathway may not participate in the *H. exemplaris* molting, but in other pathways. On the other hand, the effects of metamorphosis negative regulator juvenile hormones (JH) are well studied [22]. The downstream gene of JH signaling *Krüppel homolog-1* (*Kr-h1*) negatively regulates E75 (Li et al., 2018). Additionally, expression of *Kr-h1* inhibits ecdysone biosynthesis in prothoracic glands, where 20-E precursor ecdysone are produced. These suggests that juvenile hormones inhibit the arthropod molting pathway in both ecdysone production and signal transition. Fenoxycarb, a versatile substance that functions non-dependently against juvenile hormones that are species specific, has been used intensively as a JH analog for pathway inhibition [15, 31]. Although the conservation of signaling pathway of JH have not been identified for tardigrades, fenoxycarb functions as a JH analog for a variety of metazoan species, which implies that this may also have an effect on tardigrades. Similarly, we hypothesized that 20-E may function in tardigrades considering that the EcR/USP genes were conserved. To validate if the molting pathway regulates hatching behavior, we evaluated the effect of exposure to the ecdysteroid 20-E and juvenile hormone analog Fenoxycarb. Exposure to high concentrations of Fenoxycarb or 20-E induced un-hatched embryos, suggesting that hormone exposure may have affected embryonic development or hatching. We confirmed that the majority of the un-hatched embryos hatched after hormone removal developed claws, suggesting that exposure to 20-E and fenoxycarb regulates only hatching and not embryonic development. On the contrary, exposure of both chemicals to *R. varieornatus* did not affect hatching timing. This was not the result of the lack of expression of related genes; the expression of EcR/USP downstream genes were constitutively expressed at moderate levels in the *R. varieornatus* embryo (higher levels prior to the initial molting). These suggests that similar hatching regulation mechanisms may not be conserved in *R. varieornatus*. Additionally, previous studies have suggested that tardigrade embryos may enter a “rest state” induced by environmental perturbations [1, 2]. Although there have been no reports of a rest state induced in *H. exemplaris*, hatching of hatch-inhibited embryo after the removal of hormones is remarkably similar. These observations suggest that the embryo hatching process can be regulated by chemicals, both positively and negatively. The differences between the time of effects between chemicals may be due to the differences of incorporation rates or the expression of receptors. Observation of inhibition of embryo hatch in high concentration *H. exemplaris* exposure suggests that a portion of the chemicals are penetrating the eggshell, of which such concentrations do not affect development but only the hatch regulation mechanism.

Together, these findings suggest that the variation in embryo hatching may suggest an adaptation against their living habitat; xerophilic *R. varieornatus* inhabits a desiccation-prone environment, and hygrophilic *H. exemplaris* is less likely to face environmental desiccation on a regular basis. Therefore, *R. varieornatus* may possess variation in hatching timing in order to avoid loss of entire broods that faces environmental stress close to hatching, therefore hatching regulation by the molting pathway may not be required. On the contrary, *H. exemplaris* may allow for controlled hatching regulation through internal hormones to enhance proliferation by a more rapid development. However, we have yet to validate if the phenotypic changes to the embryo originates from the chemical exposure; the lack of phenotypic change in low concentration exposure in both species may be result of failure of chemicals to penetrate the eggshell or 20-E/Fenoxycarb may not be the proper chemicals to use as juvenile hormone analogs. Furthermore, our findings are based on only two species from two families, Hypsibiidae and Ramazzottiidae; molecular data on other species or families will be required to validate our hypothesis. We await further studies to confirm if these findings are general to the Tardigrada phylum.

## Conclusions

We conducted a comparative transcriptomic study using two tardigrade species to identify pathways induced for hatching regulation, and then validated whether these critical points could be interfered with by chemical exposure. We observed that the 3rd day of the embryo in *H. exemplaris* has a unique transcriptome profile compared to other stages, approximately 1 day prior to hatching, presumably for the strict control of its timing. Mirroring this observation, exposure of eggs to chemicals before the 3rd day influenced hatching only in *H. exemplaris*, which suggests that related pathways may be a critical component of *H. exemplaris* embryo hatching.

## Methods

### Tardigrade rearing and life history analysis

*R. varieornatus* was maintained using the methods previously described [18]. Briefly, individuals were placed on 2% Bacto agar (Difco) gels prepared with Volvic mineral water and fed with *Chlorella vulgaris* (Chlorella Industry). Individuals were moved to a new plate every week. The cultures were examined daily to obtain eggs and were observed every day to obtain juvenile samples for days 3–7.

*H. exemplaris* was also maintained using the same method with minor modifications (1.2% Bacto agar gel plates). For the life history analysis, 21 hatchlings were isolated from the culture plates, and were imaged every day. Body length was measured using ImageJ, and the days required for vitellogenesis, laying eggs days, and the number of eggs in each clutch were noted. Seventy-three eggs were used for hatching day observation. Standard variation was calculated for the hatching timing.

### Transcriptome sequencing and data preparation

Isolated *R. varieornatus* specimens were collected and were subjected to transcriptome sequencing using the methods previously described with three replicates [9]. In brief, specimens were thoroughly washed with Milli-Q water on a sterile nylon mesh (Millipore), and were placed in a low-binding PCR tube. Pipette tips were used to homogenize the animals and lysed with the TRIzol reagent (Life Technologies). Total RNA was extracted using a Direct-zol RNA kit (Zymo Research), and the quality was validated with High Sensitivity RNA ScreenTape on TapeStation 2200 (Aglient Technologies). The mRNA was amplified with the SMARTer Ultra Low Input RNA Kit for Sequencing v.3, and Illumina libraries were prepared with the KAPA HyperPlus Kit (KAPA Biosystems). The library was quantified using the Qubit Fluorometer (Life Technologies), and size distribution was validated with TapeStation D1000 ScreenTape (Agilent Technologies). The library was size selected for 200 bp by manually cutting out an agarose gel and was purified with a NucleoSpin Gel and PCR Clean-up kit (Clontech). The samples were sequenced using the NextSeq 500 High Output Mode 75 cycles kit (Illumina) as single end reads. Adaptor sequences were removed, and the reads were de-multiplexed using the bcl2fastq v. 2 software (Illumina). All mRNA-Seq data have been uploaded to GEO under the accession ID GSE130111.

### Informatics analysis

We previously performed time-series transcriptome analysis using the developmental stages of both *H. exemplaris* and *R. varieornatus* (Egg 1-5d), in addition to the juvenile stages of *H. exemplaris* (Juvenile 1-7d) and *R. varieornatus* (Juvenile 1d) [48]. These RNA-Seq data contained 4~17 M reads per sample. mRNA-Seq and small RNA-Seq data were downloaded from SRP098585 and SRX2495676, respectively. Sequence data were validated for sequencing quality using the FastQC software [5].

Amino acid and coding sequences and annotations for *R. varieornatus* and *H. exemplaris* were downloaded from www.ensembl.tardigrades.org [48]. Gene expression values were quantified using Kallisto v. 0.42.4 with the options: --bias -b 100 -t 31 --single -l 350 -s 50 [12]. The expression profiles were clustered by Self-Organizing Map (SOM, 2 × 3) using transcripts with an average over 1 TPM in all samples. To identify differentially expressed genes (DEGs), the RNA-Seq data were mapped to coding sequences with BWA MEM v. 0.7.12-r1039 [27], after summarizing mapped read counts with in-house Perl scripts, statistical tests were conducted with DESeq2 v. 1.8.2 [30]. Genes with BH method adjusted *p*-values ≦ 0.05 were defined as DEGs.

Query genes were obtained from UniProt KB [44], and were subjected to a BLASTP v2.2.22 [4] search against the amino acid sequences to determine tardigrade orthologs. For hormone receptor related gene identification, we screened the gene annotations for receptor genes that had Gene Ontology terms that contained “hormone”. Additionally, we obtained single embryo RNA-Seq data of *H. exemplaris* [26], and assembled a 3′ biased stranded transcriptome with Trinity v. 2.4.0 (default parameters). This transcriptome assembly was subjected to a BLASTn search against a previous assembly of the whole transcriptome of *H. exemplaris*, which then were further subjected to BLASTn searches against the coding sequences to identify the originating genes [48]. To quantify gene expression, the RNA-Seq data were mapped against the assembled 3′ biased stranded transcriptome with BWA MEM, and after summarizing mapped read counts with SAMtools idxstat v. 1.4 [28], TPM values were calculated in R.

### Chemical exposure

Fenoxycarb (Wako, Japan) and 20-Hydroxyecdysone (20-E, Tokyo Chemical Industry Co., Ltd., Japan) were used as juvenile hormone analog and ecdysone, respectively. Chemicals were eluted in 15 μL 100% Ethanol (Nacalai tesque, Japan), and were further diluted with 45 ml of Volvic water to obtain the exposure concentrations (20-E: 8.32 μM, 100 μM, Fenoxycarb: 3.3 μM, 100 μM) following previous research on *Daphnia* [10, 42]. For controls, embryos were exposed to 15 μl 100% ethanol in 45 ml Volvic solution (0.03% Ethanol) at day 0. Embryos (0–3 days after oviposition) with exuvium (*H. exemplaris*) or freely laid eggs (*R. varieornatus*) were exposed to the chemical solution in 12 well dishes, and the number of days required for the eggs to hatch was observed for 6 days after oviposition. *R. varieornatus* embryos were exposed to only low concentrations. We also exposed embryos to 100 μM of ecdysone receptor antagonist Cucurbitacin B (Tokyo Chemical Industry Co., Ltd., Japan) to assess whether inhibiting the molting pathway affected embryo hatching [45, 50]. We assayed six and three clutches for low and high concentration exposures, respectively. Three clutches were assayed for the 100 μM Cucurbitacin B treatment. For *R. varieornatus*, we assayed three clutches for both chemicals. Treated samples (including control samples) were incubated at 18 °C. Furthermore, to validate if unhatched embryos were capable of hatching, embryos were washed with Volvic water three times to remove chemicals and then cultured until hatching could be confirmed. To assess whether the unhatched embryo had completed development, the cuticle shells of embryos that did not hatch at Egg 7d were peeled off with a 26G needle and were observed with light microscopy for claw formation.

### Statistics

All statistics analysis were conducted in the R software. Standard deviation for each data are shown throughout this manuscript. Heatmaps and line plos were generated using R and ggplot2 library.

## Supplementary information

**Additional file 1:** **Figure S1.** Body length in developing individuals of *H. exemplaris.* The body length was quantified for new hatchlings and observed for 28 days.

**Additional file 2:** **Figure S2.** HOX genes were upregulated around Egg 2-3d in both species. Z-scaled TPM values of HOX genes identified in Yoshida et al. [48] were visualized as a heatmap. E: Egg, B: Juvenile, Active/Tun: Adult stages.

**Additional file 3:** **Figure S3.** Expression of HOX genes in the CEL-Seq data set. CEL-Seq reads were assembled to construct a 3′ strand biased transcriptome, used to identify HOX genes. The CEL-Seq reads were mapped against this assembly to calculate gene expression (TPM).

**Additional file 4:** **Figure S4.** Expression profiles of CAHS and SAHS orthologs. TPM values of (A) CAHS and (B) SAHS genes were plotted with standard deviation as error bars.

**Additional file 5:** **Figure S5.** Profiles of anti-oxidative stress related genes. Z-scaled TPM values of catalase (CATE), glutathione S-transferase (GST), superoxide dismutase (SOD), and Thioredoxin reductase (TRX) of both species were plotted as a heatmap.

**Additional file 6:** **Figure S6.** Duration of *H. exemplaris* embryo exposed to low concentration chemicals. Days required for hatching in embryo exposed to low concentrations. NH: Not hatched. Error bars indicates standard variation.

**Additional file 7:** **Figure S7.** Exposure to low concentration chemicals in both tardigrades. Embryo were exposed to low concentrations of Fenoxycarb and 20-E at the indicated days, and the hatching ratio was recorded. Slightly higher variation was observed in the H. exemplaris embryos, but not in *R. varieronatus.*

**Additional file 8:** **Figure S8.** Duration of *H. exemplaris* embryogenesis exposed to high-concentration chemicals. Days required for hatching in embryo exposed to high concentrations. NH: Not hatched. Error bars indicates standard variation.

**Additional file 9:** **Table S1.** Summary of RNA-Seq data.

**Additional file 10. **Expression profiles of genes in group 1 and 2 of SOM clustering in *H. exemplaris*. https://github.com/abs-yy/Yoshida_and_Sugiura_etal_2018/blob/master/Sdata_1_hypsibius_som_group1ANND2.xlsx.

**Additional file 11.** Excel file containing the expression profiles of genes focused in this analysis. https://github.com/abs-yy/Yoshida_and_Sugiura_etal_2018/blob/master/Sdata_2_geneexpression.xlsx.

**Additional file 12.** Annotations of genes overlapping piRNA loci predicted in both PILFER and proTRAC. https://github.com/abs-yy/Yoshida_and_Sugiura_etal_2018/blob/master/piRNA_clusters_intersect_geneAnnotations.txt.

**Additional file 14.** Additional text on the analysis pf piRNA genes and anhydrobiosis related genes.

## Data Availability

All mRNA-Seq data have been uploaded to GEO under the accession ID GSE130111.

## Abbreviations

20-E: 20-Hydroxyecdysone
ANOVA: Analysis of Variance
EcR: Ecdysone receptor
hpl: hours post laying
JH: Juvenile hormones
Kr-h1: Krüppel homolog-1
SD: Standard deviation
SEM: Scanning Electron Microscope
SOM: Self-organizing map
Tukey HSD: Tukey Honestly Significant Difference
USP: Ultraspiracle
