# Supplementary figures and images for: Comparison of the transcriptomes of two tardigrades with different hatching coordination
Source: BMC Dev Biol. 2019 Dec 9;19:24. doi: 10.1186/s12861-019-0205-9 (PMC6925440; doi:10.1186/s12861-019-0205-9)

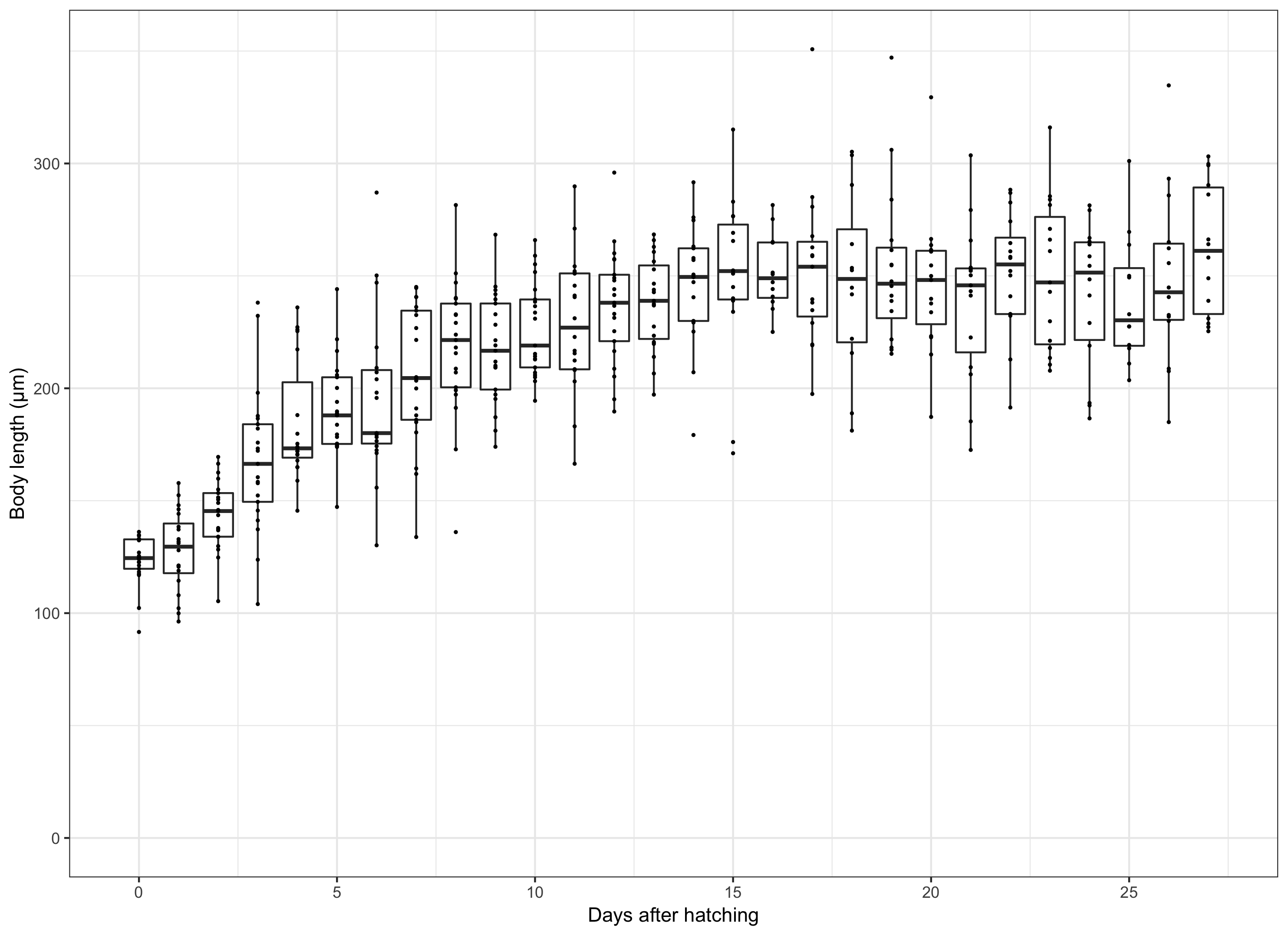

Supplement: Supplementary file 2 — Additional file 1: Figure S1. Body length in developing individuals of H. exemplaris. The body length was quantified for new hatchlings and observed for 28 days. [file 12861_2019_205_MOESM1_ESM.png]

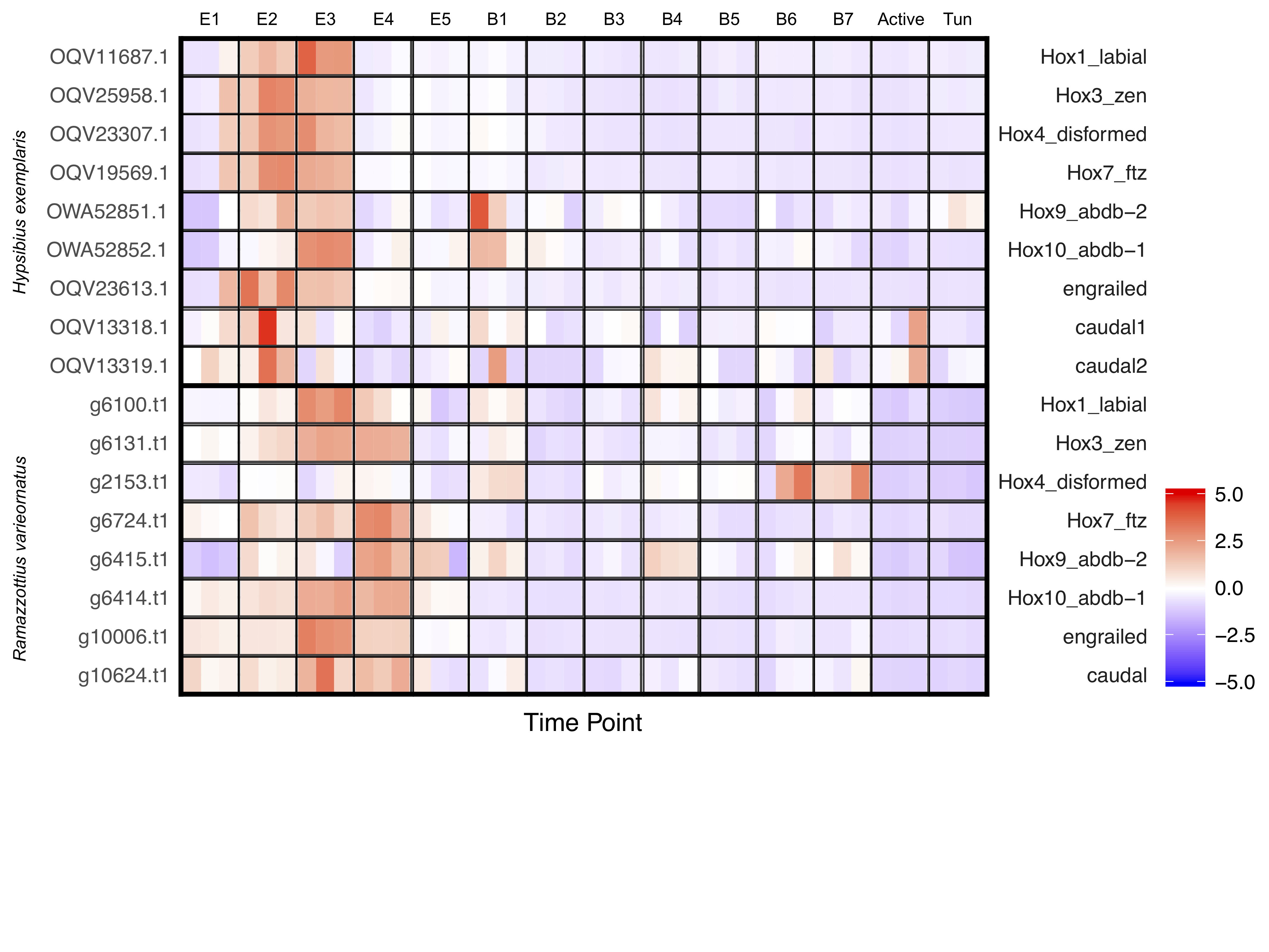

Supplement: Supplementary file 3 — Additional file 2: Figure S2. HOX genes were upregulated around Egg 2-3d in both species. Z-scaled TPM values of HOX genes identified in Yoshida et al. [48] were visualized as a heatmap. E: Egg, B: Juvenile, Active/Tun: Adult stages. [file 12861_2019_205_MOESM2_ESM.tiff]

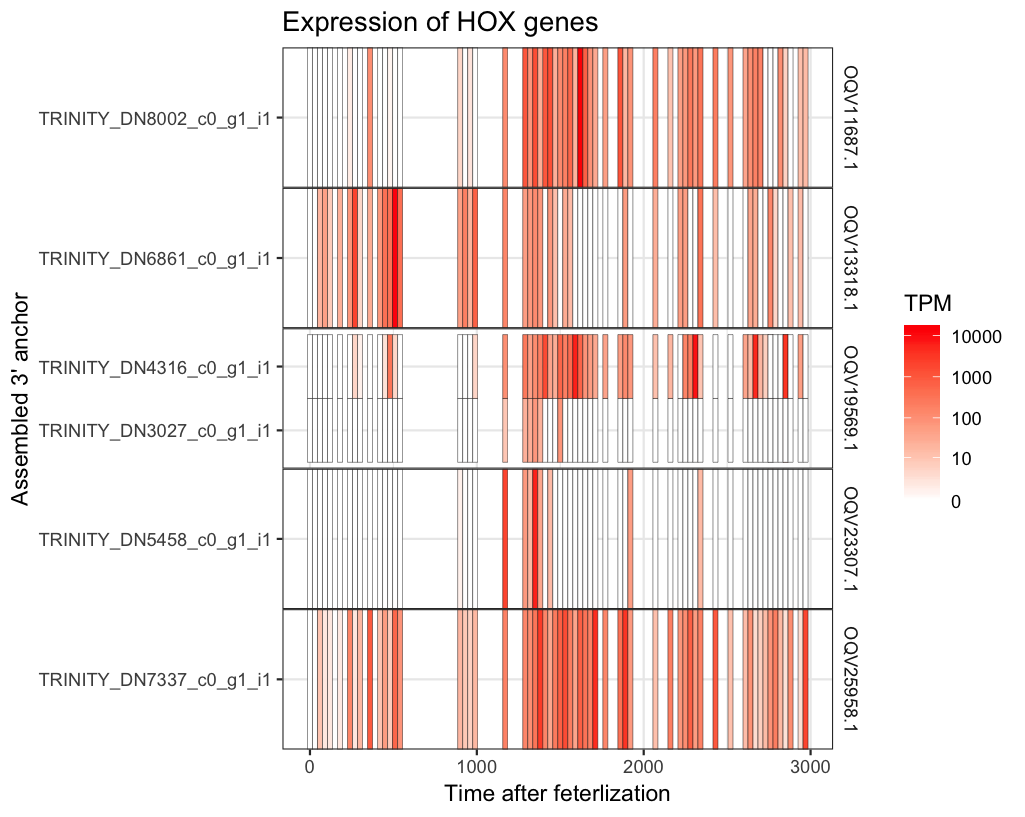

Supplement: Supplementary file 4 — Additional file 3: Figure S3. Expression of HOX genes in the CEL-Seq data set. CEL-Seq reads were assembled to construct a 3′ strand biased transcriptome, used to identify HOX genes. The CEL-Seq reads were mapped against this assembly to calculate gene expression (TPM). [file 12861_2019_205_MOESM3_ESM.png]

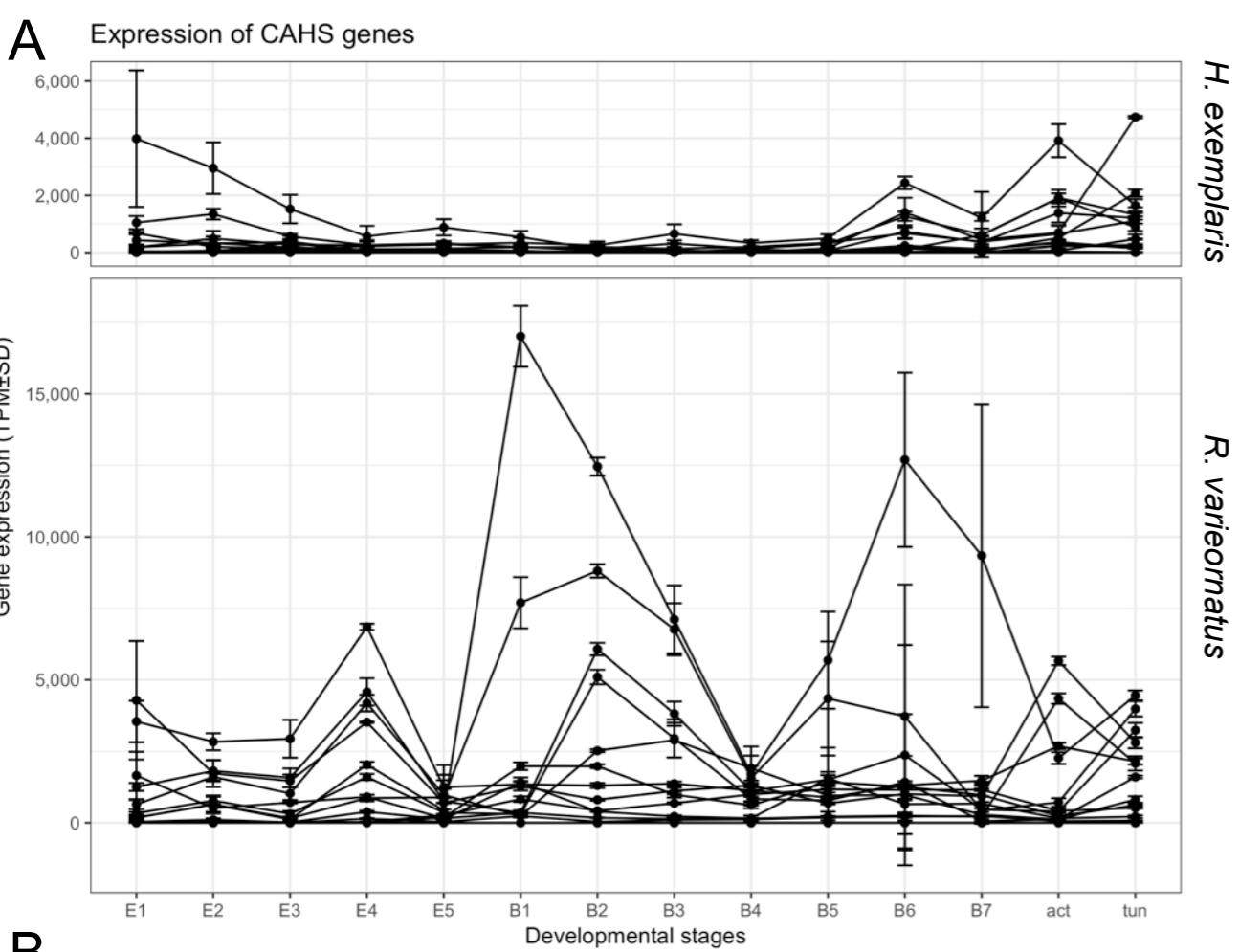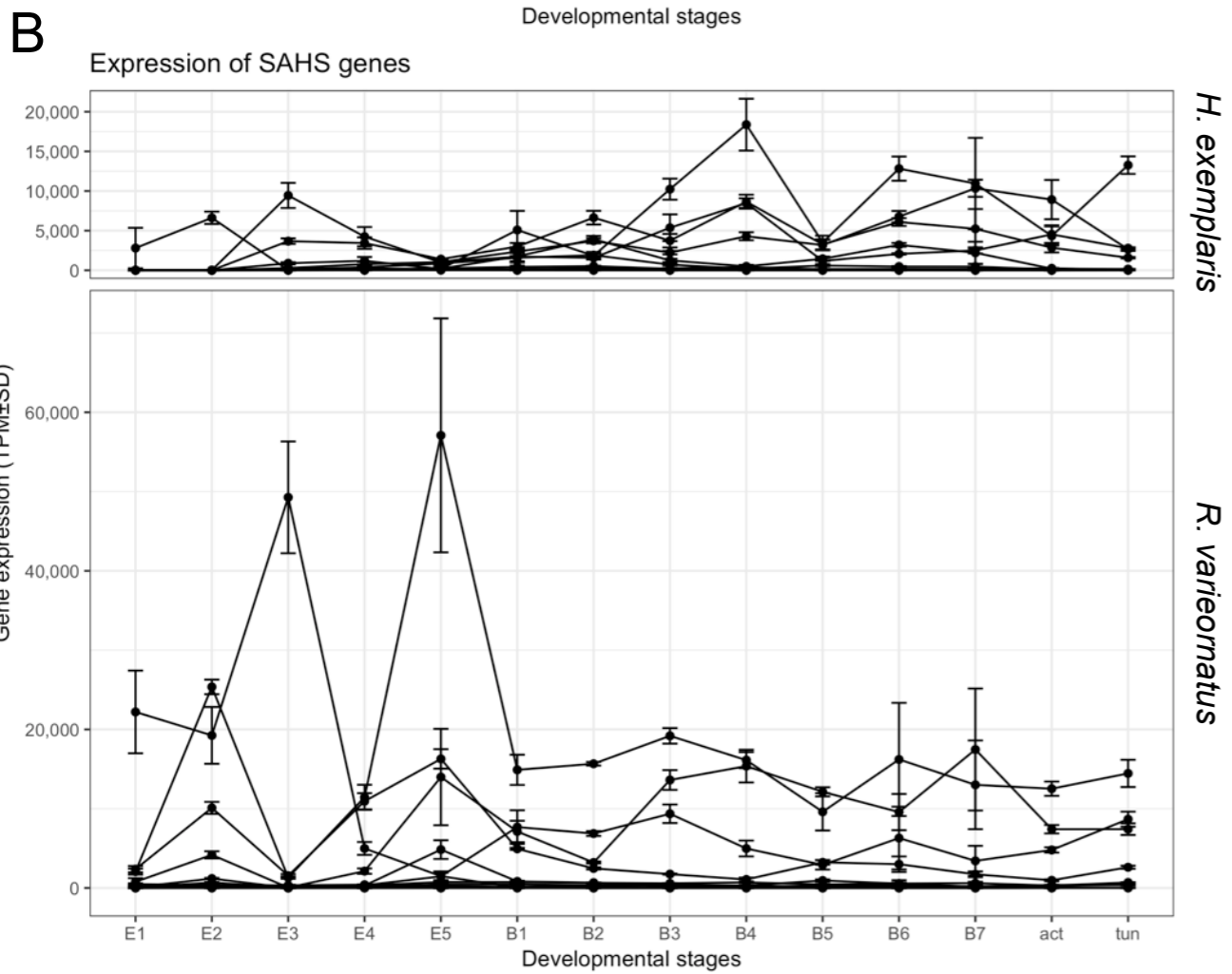

Supplement: Supplementary file 5 — Additional file 4: Figure S4. Expression profiles of CAHS and SAHS orthologs. TPM values of (A) CAHS and (B) SAHS genes were plotted with standard deviation as error bars. [file 12861_2019_205_MOESM4_ESM.pdf]

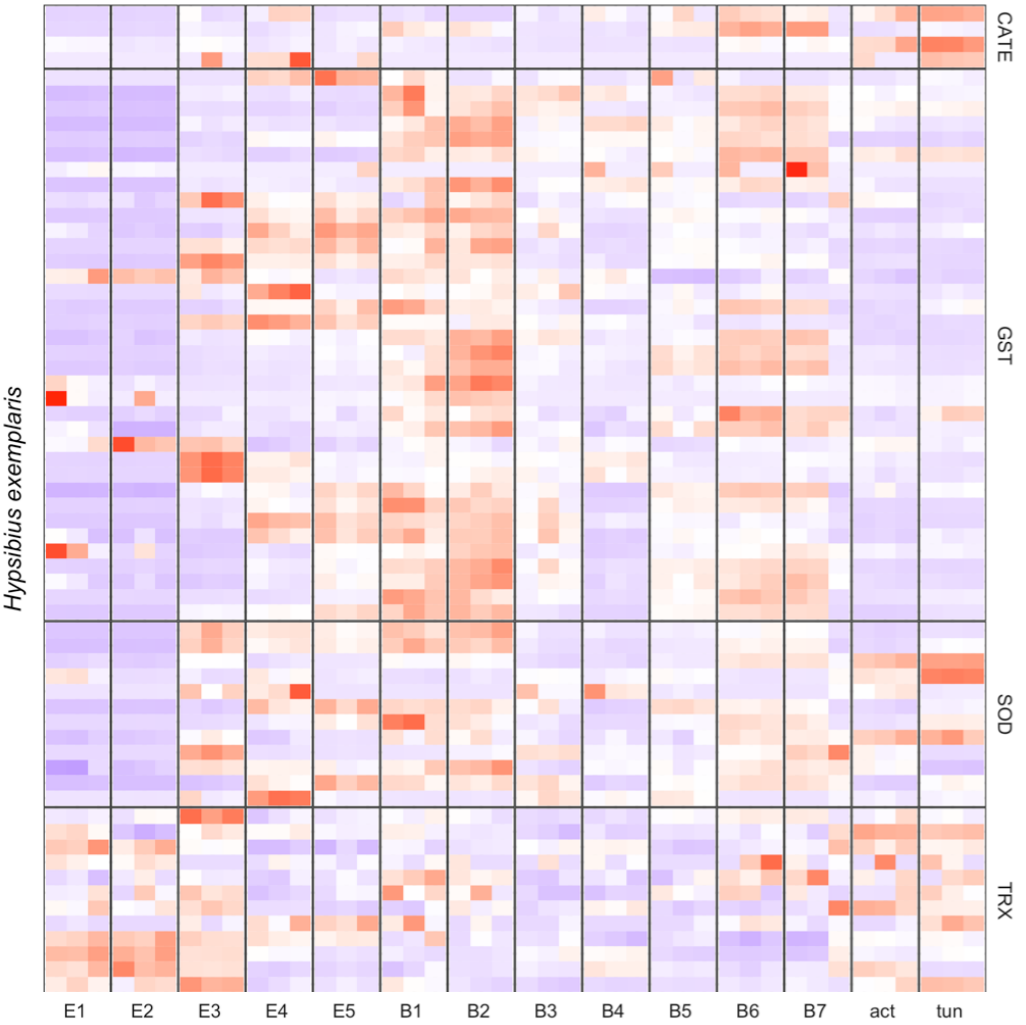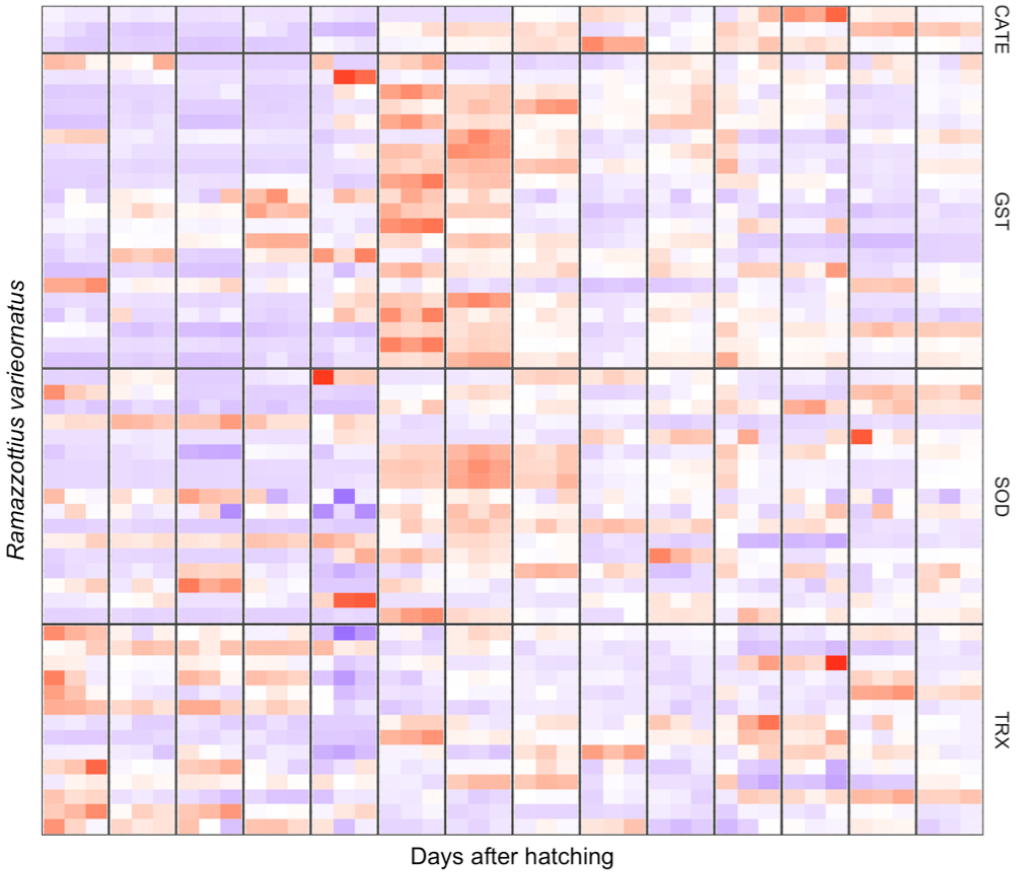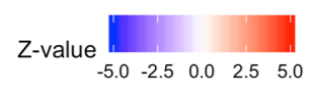

Supplement: Supplementary file 6 — Additional file 5: Figure S5. Profiles of anti-oxidative stress related genes. Z-scaled TPM values of catalase (CATE), glutathione S-transferase (GST), superoxide dismutase (SOD), and Thioredoxin reductase (TRX) of both species were plotted as a heatmap. [file 12861_2019_205_MOESM5_ESM.pdf]

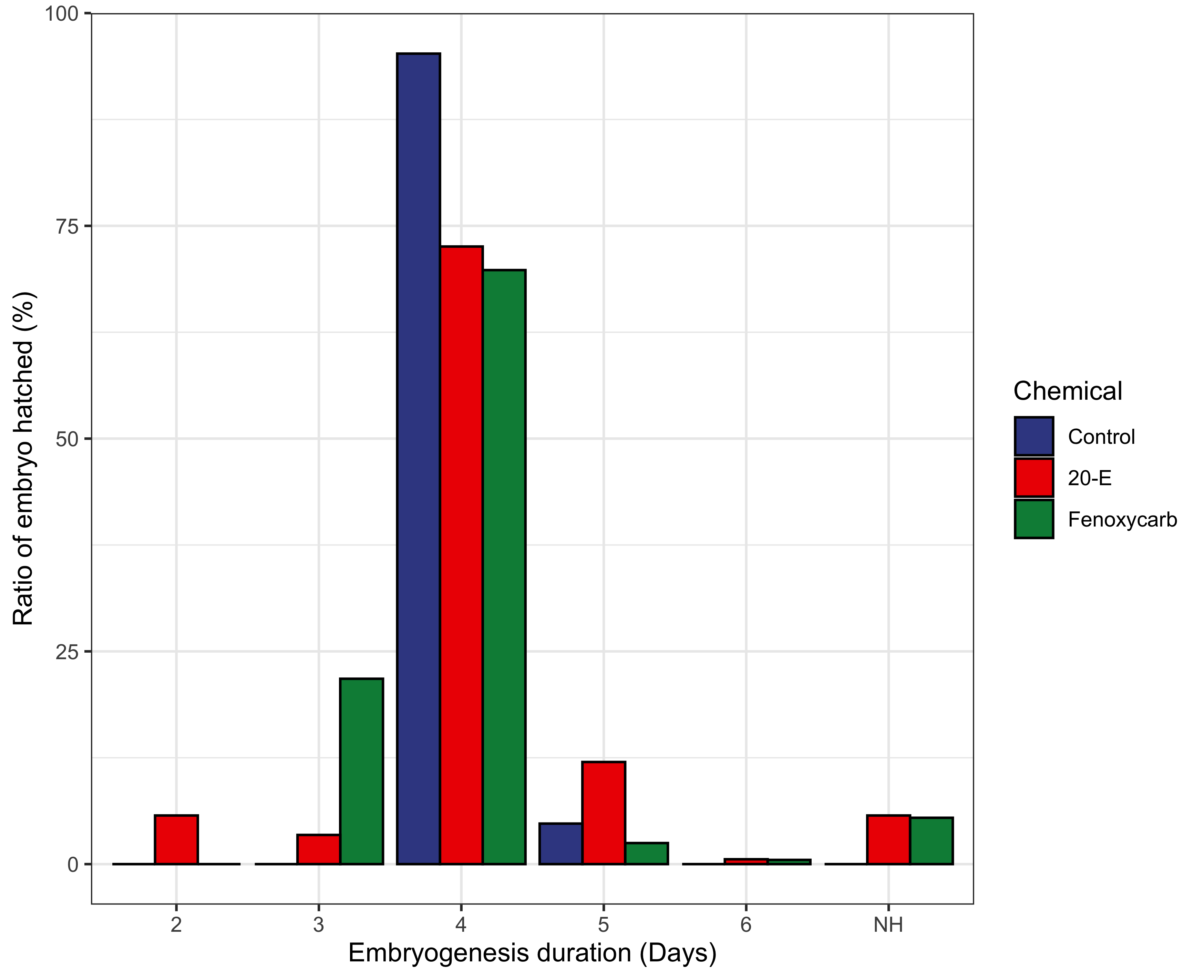

Supplement: Supplementary file 7 — Additional file 6: Figure S6. Duration of H. exemplaris embryo exposed to low concentration chemicals. Days required for hatching in embryo exposed to low concentrations. NH: Not hatched. Error bars indicates standard variation. [file 12861_2019_205_MOESM6_ESM.png]

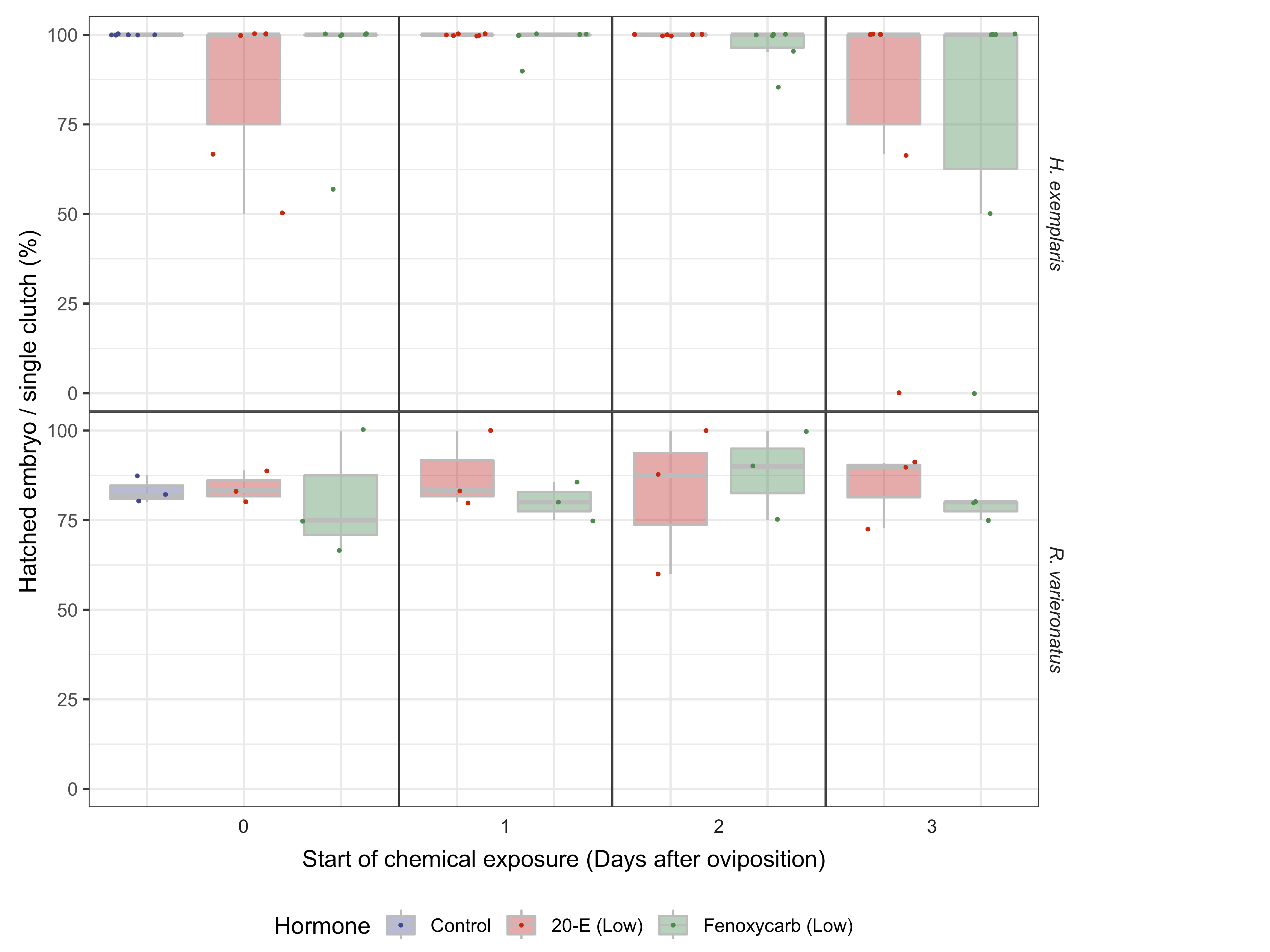

Supplement: Supplementary file 8 — Additional file 7: Figure S7. Exposure to low concentration chemicals in both tardigrades. Embryo were exposed to low concentrations of Fenoxycarb and 20-E at the indicated days, and the hatching ratio was recorded. Slightly higher variation was observed in the H. exemplaris embryos, but not in R. varieronatus. [file 12861_2019_205_MOESM7_ESM.png]

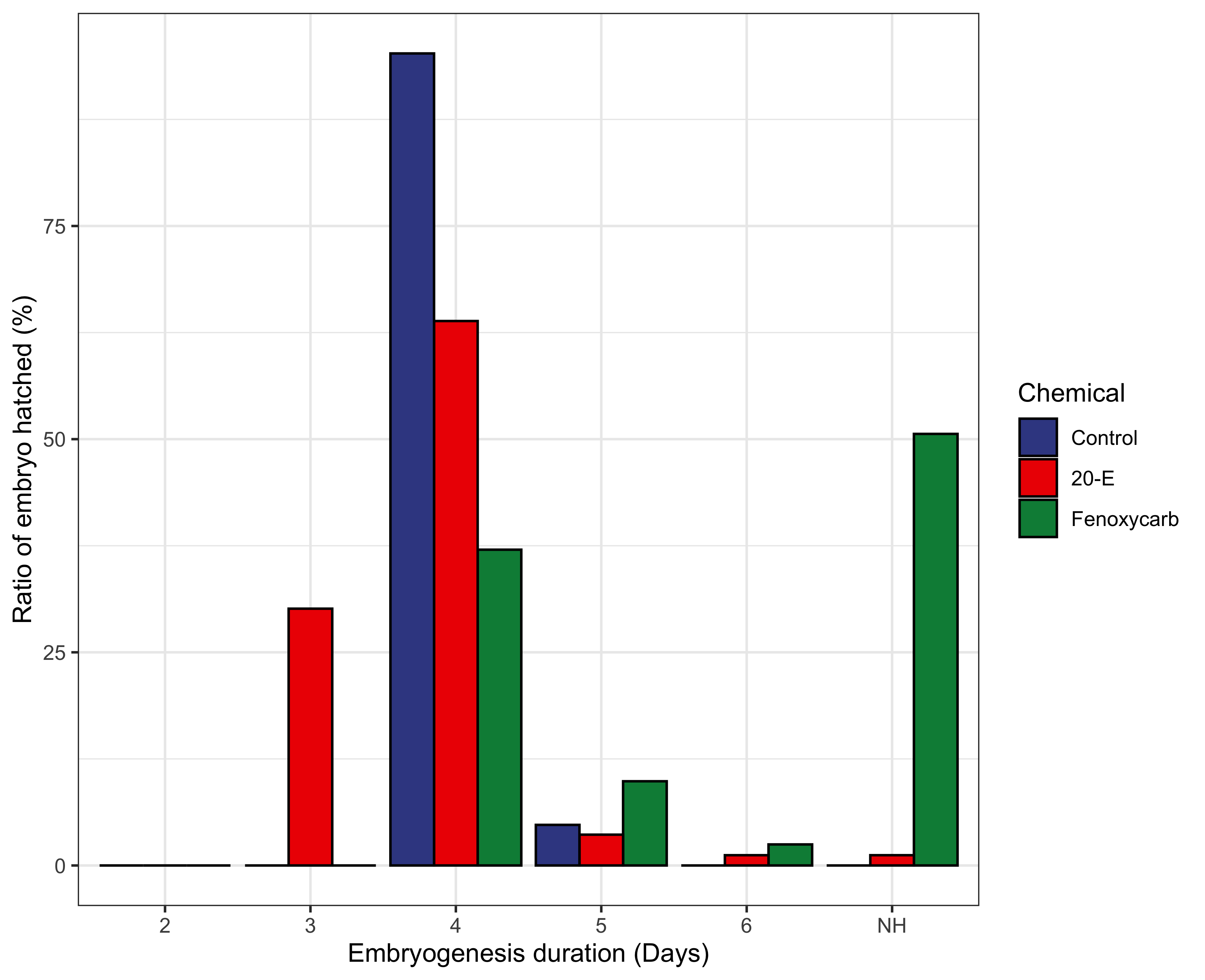

Supplement: Supplementary file 9 — Additional file 8: Figure S8. Duration of H. exemplaris embryogenesis exposed to high-concentration chemicals. Days required for hatching in embryo exposed to high concentrations. NH: Not hatched. Error bars indicates standard variation. [file 12861_2019_205_MOESM8_ESM.png]
