# Supplementary material for: Comparison of the transcriptomes of two tardigrades with different hatching coordination
Source: BMC Dev Biol. 2019 Dec 9;19:24. doi: 10.1186/s12861-019-0205-9 (PMC6925440; doi:10.1186/s12861-019-0205-9)
Supplement: Supplementary file 14 — Additional file 14. Additional text on the analysis pf piRNA genes and anhydrobiosis related genes. [file 12861_2019_205_MOESM14_ESM.docx]

1. **piRNA genes**

To identify piRNA clusters, we used our previously sequenced small RNA-Seq data (Yoshida et al., 2017). Sequenced reads were mapped to known non-coding RNA sequences of *H. exemplaris* (tRNA, rRNA, miRNA from Yoshida et al. (2017)), and reads that were not mapped were used as follows. After removal of redundant and low-complexity reads using scripts from proTRAC (Rosenkranz and Zischler, 2012), we mapped the remaining reads to piRBase (Wang et al., 2019) to identify piRNA sequences from *C. elegans* and *D. melanogaster*. The reads that did not map to piRBase were further mapped to the *H. exemplaris* genome, and proTRAC v. 2.4.2 and PILFER (downloaded at 2018.11.05) were used for piRNA cluster identification (Ray and Pandey, 2018; Rosenkranz and Zischler, 2012). File conversion was conducted using BEDtools v. 2.25.0, SAMtools and G-language Genome Analysis Environment v 1.9.1 (Arakawa et al., 2010; Arakawa et al., 2003; Arakawa and Tomita, 2006; Li et al., 2009; Quinlan and Hall, 2010), and read mapping was conducted using Bowtie2 v. 2.2.9 (Langmead and Salzberg, 2012).

We observed that the expression of small RNA-related genes (Argonaute, OQV17169.1/ OQV21162.1, g3665.t1) was induced at the developmental stages, which supports the implication of piRNA existence suggested in previous studies. We used our previously sequenced small RNA-Seq data and observed that 1.5M reads mapped to the *H. exemplaris* genome, but only 27K reads to known piRNA sequences from *C. elegans* and *D. melanogaster* in piRbase. Using these piRNA candidates, PILFER and proTRAC, predicted 894 and 187 piRNA clusters, respectively (Yoshida et al. 2019). We designated the 201 regions that were identified in both methods as putative piRNA clusters in *H. exemplaris*, which overlapped with 490 genes (504 transcripts), of which 385 transcripts were hypothetical genes (Yoshida et al. 2019, **Supplementary Data S3**).

Using our previously sequenced *H. exemplaris* small RNA-Seq data　(Yoshida et al., 2017), we predicted piRNA clusters in the *H. exemplaris* genome, consistent with previous studies (Sarkies et al., 2015). Most of these piRNA clusters showed no homology with *D. melanogaster* nor *C. elegans* piRNAs, suggesting most of these piRNAs may have been acquired after divergence from the common ancestors of Arthropoda and Nematoda.

1. **Anhydrobiosis related genes**

We observed that several copies of CAHS and SAHS genes were expressed exclusively in the developmental stages (**Supplementary Figure S4AB**, **Supplementary Table S2**). Additionally, we have found that the anti-oxidative stress-related thioredoxin was also induced in Egg 3d in both species (**Supplementary Figure S5**). In comparison, glutathione S-transferase and superoxide dismutase were most expressed during the early juvenile stages, and catalase at late juvenile to adult stages.

Tardigrades are famous for their tolerance capabilities, such as desiccation tolerance (Keilin, 1959). Although we found several CAHS and SAHS orthologs to have relatively high expression during the embryonic stages in both species (Yamaguchi et al., 2012), we found one SAHS ortholog that showed particularly high expression in both embryonic stages, suggesting that the regulation of an embryo-specific ortholog may be conserved between Hypsibiidae and Ramazzottiidae. Along with the expression profiles of antioxidative genes, these data suggest the fundamental anhydrobiosis mechanism during the embryonic stages in Hypsibiidae and Ramazzottiidae. Additional analysis of the developmental stages in *Milnesium inceptum*, a terrestrial species capable of anhydrobiosis in the embryonic stages (Schill and Fritz, 2008), may further support our findings.

Arakawa, K., Kido, N., Oshita, K., Tomita, M., 2010. G-language genome analysis environment with REST and SOAP web service interfaces. Nucleic Acids Res 38, W700-705.

Arakawa, K., Mori, K., Ikeda, K., Matsuzaki, T., Kobayashi, Y., Tomita, M., 2003. G-language Genome Analysis Environment: a workbench for nucleotide sequence data mining. Bioinformatics 19, 305-306.

Arakawa, K., Tomita, M., 2006. G-language system as a platform for large-scale analysis of high-throughput omics data. J. Pestic. Sci. 31, 282-288.

Langmead B, Salzberg SL. Fast gapped-read alignment with bowtie 2. Nat Methods. 2012;9:357–9.

Quinlan, A.R., Hall, I.M., 2010. BEDTools: a flexible suite of utilities for comparing genomic features. Bioinformatics 26, 841-842.

Ray, R., Pandey, P., 2018. piRNA analysis framework from small RNA-Seq data by a novel cluster prediction tool - PILFER. Genomics 110, 355-365.

Rosenkranz, D., Zischler, H., 2012. proTRAC--a software for probabilistic piRNA cluster detection, visualization and analysis. BMC Bioinformatics 13, 5.

Sarkies, P., Selkirk, M.E., Jones, J.T., Blok, V., Boothby, T., Goldstein, B., Hanelt, B., Ardila-Garcia, A., Fast, N.M., Schiffer, P.M., Kraus, C., Taylor, M.J., Koutsovoulos, G., Blaxter, M.L., Miska, E.A., 2015. Ancient and novel small RNA pathways compensate for the loss of piRNAs in multiple independent nematode lineages. PLoS Biol 13, e1002061.

Schill, R.O., Fritz, G.B., 2008. Desiccation tolerance in embryonic stages of the tardigrade. Journal of Zoology 276, 103-107.

Wang, J., Zhang, P., Lu, Y., Li, Y., Zheng, Y., Kan, Y., Chen, R., He, S., 2019. piRBase: a comprehensive database of piRNA sequences. Nucleic Acids Res 47, D175-D180.
